# The K526R substitution in viral protein PB2 enhances the effects of E627K on influenza virus replication

**DOI:** 10.1038/ncomms6509

**Published:** 2014-11-20

**Authors:** Wenjun Song, Pui Wang, Bobo Wing-Yee Mok, Siu-Ying Lau, Xiaofeng Huang, Wai-Lan Wu, Min Zheng, Xi Wen, Shigui Yang, Yu Chen, Lanjuan Li, Kwok-Yung Yuen, Honglin Chen

**Affiliations:** 1 State Key Laboratory for Emerging Infectious Diseases, Department of Microbiology, and the Research Center of Infection and Immunology, The University of Hong Kong, Hong Kong SAR, China; 2 The Collaborative Innovation Center for Diagnosis and Treatment of Infectious Diseases, Zhejiang University, Hangzhou 310003, China; 3 State Key Laboratory for Diagnosis and Treatment of Infectious Diseases, Zhejiang University, Hangzhou 310003, China

## Abstract

Host-adaptive strategies, such as the E627K substitution in the PB2 protein, are critical for replication of avian influenza A viruses in mammalian hosts. Here we show that mutation PB2-K526R is present in some human H7N9 influenza isolates, in nearly 80% of H5N1 human isolates from Indonesia and, in conjunction with E627K, in almost all seasonal H3N2 viruses since 1970. Polymerase complexes containing PB2-526R derived from H7N9, H5N1 or H3N2 viruses exhibit increased polymerase activity. PB2-526R also enhances viral transcription and replication in cells. In comparison with viruses carrying 627K, H7N9 viruses carrying both 526R and 627K replicate more efficiently in mammalian (but not avian) cells and in mouse lung tissues, and cause greater body weight loss and mortality in infected mice. PB2-K526R interacts with nuclear export protein and our results suggest that it contributes to enhance replication for certain influenza virus subtypes, particularly in combination with 627K.

## Abstract (web summary)

Mutations in the viral polymerase, such as PB2-E627K, contribute to adaptation of avian influenza strains to mammalian hosts. Here the authors show that another mutation, PB2-K526R, is found in seasonal H3N2 and avian-origin human influenza isolates, and facilitates replication of these viruses in mammals.

## Graphical abstract

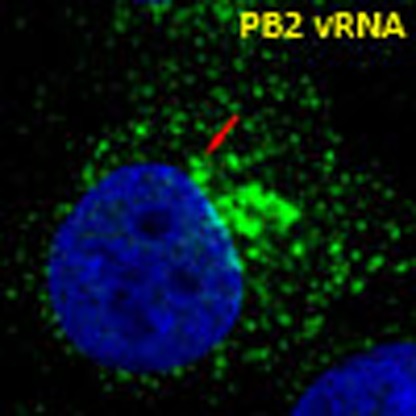

Influenza viruses utilize the viral polymerase complex, which is composed of PB1, PB2 and PA subunits, to replicate and transcribe the viral genome in the cell nucleus. Adaptation of viral polymerase is critical for efficient virus replication in a new host following cross-species transmission^1^. Several adaptation markers in the polymerase have been identified among seasonal influenza viruses that are circulating in humans and avian influenza viruses, which cause sporadic human infections^1^,^2^. The most well-characterized adaptation marker is PB2 E627K, which was found in a significant proportion of H5N1 human infections and also in one case of H7N7 infection^3^,^4^. However, 627K is not exclusive to all influenza viruses that can replicate in humans^5^,^6^. Another adaption marker, PB2 590S/591R, was reported to enable replication fitness of swine origin 2009 H1N1 virus in humans^7^,^8^. PB2 D701N and Q591K substitutions were also found to support H5N1 virus replication in mammalian hosts^8^,^9^. The E627K substitution enhances H5N1 virus replication in the upper respiratory tract, where the temperature, at 33 °C, is slightly lower than the core body temperature^8^. PB2 627K and 627E/701N have been associated with enhanced transmission of influenza virus in mammalian hosts^10^. A T271A substitution in PB2 was also described to enhance replication of 2009 H1N1 and H5N1 viruses in mammalian cells *in vitro*^11^,^12^,^13^. PB2 has been reported to interact with host α-importins; it is thought that differential binding of PB2 with importin-α1 and -α7 may regulate influenza virus polymerase activity^14^,^15^. Details of how these genetic substitutions translate into adaptive mechanisms remain largely unknown.

While about 50% of H5N1 human isolates contain known PB2 adaptation markers, mainly E627K with some D701N and a few instances of 591K, as described above, the other half of human infections are not associated with known adaptation markers^16^,^17^,^18^. Indonesia has experienced the highest number of human H5N1 infections, with 195 cases, 163 of which were fatal (http://www.who.int/influenza/human_animal_interface/EN_GIP_20140124CumulativeNumberH5N1cases.pdf). However, the majority (80%) of the H5N1 isolates from human cases in Indonesia do not harbour the PB2 627K or 701N markers. Apart from the markers described above, influenza viruses probably utilize uncharacterized adaptive substitutions in the PB2 polymerase subunit to support virus replication in human cells. In the spring of 2013, a novel avian H7N9 virus emerged that crossed the species barrier to infect humans in eastern China^19^,^20^. More than 300 laboratory-confirmed cases of human infection, about 30% of which were fatal, have been identified since March 2013. While sporadic human infections caused by avian H5N1, H9N2 and H7N7 viruses have been recorded since 1997 (refs 21, 22, 23), no avian influenza virus has caused an outbreak of this magnitude since the 1918 pandemic^24^. It is important to understand how this H7N9 virus gained the ability to infect humans so easily. Genetic characterization revealed that the H7N9 virus is a reassortant virus, possibly generated through multiple reassortment events to incorporate the haemagglutinin (HA) gene from an H7N3 virus, the neuraminidase gene originating from an H7N9 virus circulating in wild birds and other internal genes from H9N2 virus present in poultry^19^,^20^,^25^. Genetic and structural analyses found that the HA of H7N9 contains a Q226L substitution, which may confer a certain level of ability for binding to human type sialic acid receptors^26^,^27^. However, the Q226L mutation has been found in avian H9N2 viruses, which have been prevalent in poultry for several years, with only a limited number of human infections having been identified since 1997 (ref. 28). There is strong interest in understanding what other features of 2013 H7N9 virus facilitate its infection of humans.

This study analyzes influenza sequences available from public databases and also those arising from our analysis of H7N9 isolates from human cases to identify new adaptation marker(s) in the PB2 gene. Various PB2 adaptation markers are identified among the H7N9 human isolates, and a K526R substitution associated with other previously defined adaptive markers is detected in some human isolates. Further characterization of the PB2-526R genotype reveals that H7N9 virus carrying this marker replicates to a significantly higher titre in mammalian cells, and also in mice. Furthermore, we show that nearly 80% of H5N1 human isolates from Indonesia and almost all seasonal H3N2 influenza A viruses isolated since 1970 contain PB2-526R. Reverse mutation of 526R to 526K attenuates polymerase activity in H3N2 and Indonesia-subclade H5N1 viruses. Therefore, in addition to 627K, 701N, 590S/591R and 591K, 526R is another marker of mammalian adaption by avian influenza viruses, possibly functioning through interaction between viral PB2 and nuclear export protein (NEP) during virus replication.

## Results

### PB2-K526R is present in three influenza virus subtypes

**Figure 1:**
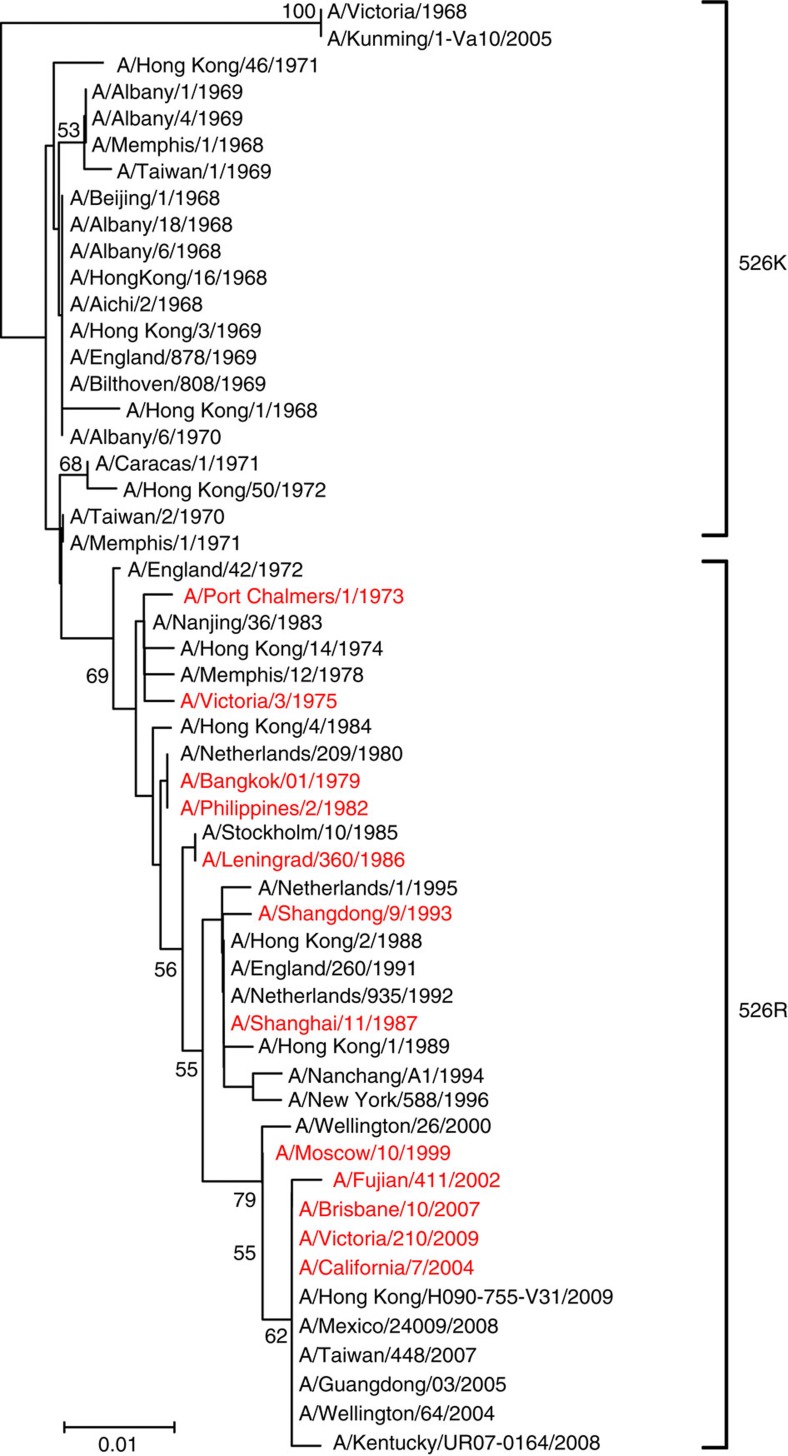
Phylogenetic tree of H3N2 virus PB2. The PB2 tree was constructed using the neighbour-joining method with the Poisson model of amino-acid substitution; the reliability of the phylogenetic tree was determined by bootstrap analysis using 1,000 replicates. All sequences were obtained from Genbank. Viruses in red represent vaccine strains recommended by the World Health Organization.

Examination of the sequences of H7N9 human isolates has revealed a variety of different mammalian adaptation markers present in PB2 genes^19^,^20^,^29^,^30^. Besides the previously described 627K, 701N and 591K loci^19^,^29^,^30^, we found a K526R genotype associated with either 627E/627K or 701D/701N (Supplementary Fig. 1). Subsequent analysis found three distinct major PB2 genotypes, 526R/627K, 526R/701N and 526R alone, to be present within some isolates obtained from our own study and in some reported H7N9 human cases identified since March 2013 in China (Table 1)^20^,^31^,^32^. The 526R substitution has not previously been described to be a mammalian adaptation marker for avian influenza viruses. However, screening of sequences in the GenBank influenza database revealed that K526R is present in some H5N1 human isolates, particularly those from human cases in Indonesia (Table 1). This may explain why the majority of human isolates from Indonesia do not carry the PB2-627K marker that is commonly found among human infections in other H5N1-affected areas. Furthermore, sequence analysis found that the H3N2 virus obtained K526R around 1970 and that the PB2-526R genotype immediately became the dominant circulating H3N2 in humans (Fig. 1; Table 1), which has continued to this day. Since H3N2 virus has been associated with humans for nearly 50 years, it seems that 526R may be a previously unidentified adaptation marker of the polymerase complex of influenza virus with the potential to become established in a viral strain, and may act alone, or in coordination with other markers, to facilitate viral replication in humans.

### PB2-526R enhances polymerase activity alone and with 672K

**Figure 2:**
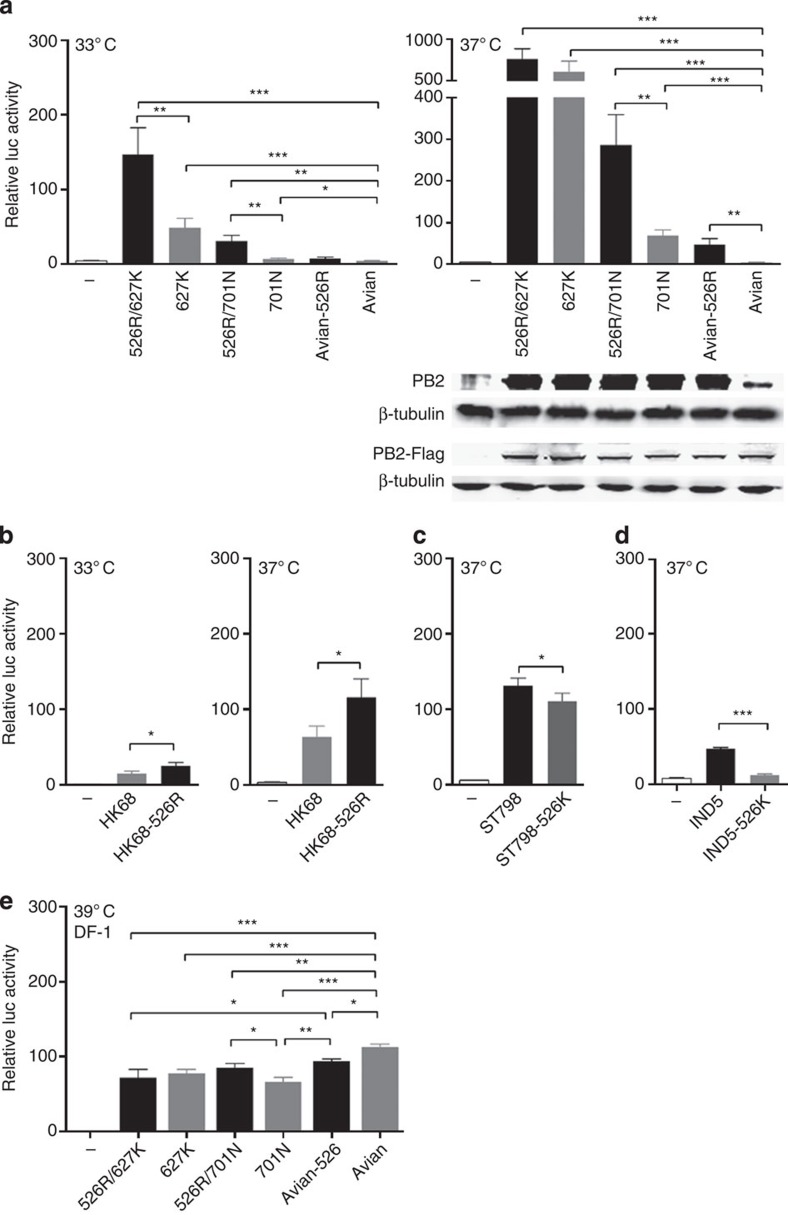
**Polymerase activity of H7N9 PB2 variants** ***in vitro***. (**a**) HEK293T cells were transfected with H7N9 RNP complexes composed of NP, PB1 and PA from the A/Zhejiang/DTID-ZJU01/2013 strain and PB2 from different strains containing different adaptation markers, as shown in Table 1, together with the firefly luciferase reporter pYH-Luci and a *Renilla* luciferase reporter (internal control). Luciferase activity was measured at 24 h post transfection, following incubation at 33 or 37 °C. Comparison of RNP polymerase activity with PB2 derived from human and avian H7N9 isolates; PB2 protein levels estimated by western blot for the minigenome assay and FLAG-tagged pCMV clones are shown at the bottom. (**b**) Comparison of RNP polymerase activity for H3N2 (A/Hong Kong/1/68) RNP containing PB2 with or without the 526R substitution in HEK293T cells at 24 h post transfection. (**c**) Comparison of RNP activity of H3N2 (A/Guangdong/ST798/2008) RNP containing PB2 with or without the 526K reverse mutation in HEK293T cells at 24 h post transfection. (**d**) Comparison of RNP activity with H5N1 (A/Indonesia/5/2005) RNP containing PB2-526R or PB2-526K reverse mutation in HEK293T cells at 24 h post transfection. (**e**) DF-1 cells were transfected with the same set of RNP complexes as in **a**. Luciferase activity was measured at 24 h post transfection, following incubation at 39 °C. Data represent mean luciferase activity from three separate experiments, calculated after normalization with *Renilla* luciferase activity,±s.d. ‘—’ represents blank control; RNP without the PB2 gene. Statistical significance was analysed by one-way analysis of variance, corrected by the Bonferroni post-test: ***P<0.001, **P<0.01 and *P<0.05. Full-size uncropped western blots showing PB2 protein levels are displayed in Supplementary Fig. 6.

**Figure 3:**
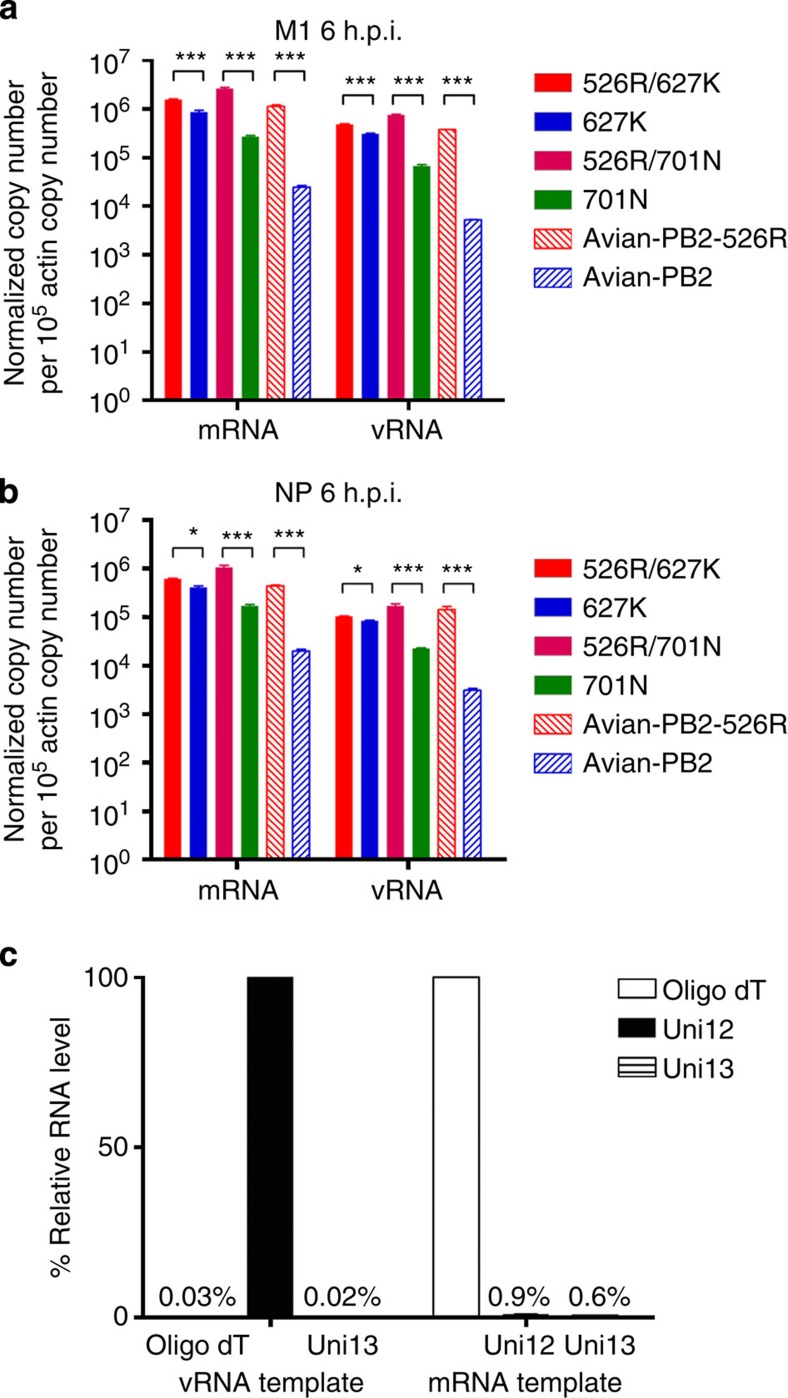
Quantitative estimation of vRNA and mRNA levels in H7N9 virus-infected A549 cells. A549 cells were infected with H7N9 viruses carrying different PB2 adaptation markers, as indicated, at an MOI of 1. Uni-12, Uni-13 and oligo dT primers were used for reverse transcription of vRNA, cRNA and mRNA, respectively. Levels of M1 (**a**) and NP (**b**) genes were estimated by quantitative RT-PCR and normalized with the β-actin gene. (**c**) Verification of specificity of primers used for viral mRNA and vRNA using oligo dT, or uni-12 or uni-13 primed cDNA from NP plasmid transfection and viral particles, respectively, as described in the Methods. The values displayed represent the mean copy number per 10^5^ β-actin copy number±s.d. from three separate experiments. Statistical significance was calculated by the *t*-test: ***P<0.001 and *P<0.05. h.p.i.: hours post infection.

The influenza A virus viral polymerase complex, which is composed of PB1, PB2 and PA subunits, is used to replicate and transcribe the viral genome in the cell nucleus. We tested whether the presence of 526R, either alone or in combination with other adaptation markers, altered the ribonucleoprotein (RNP) complex polymerase activity of H7N9 virus. We first compared polymerase activity using a minigenome assay, testing all PB2 genotypes identified from H7N9 viruses for which sequence data had been publicly released or obtained from our analyses (Table 1). As expected, PB2 carrying 627K or 701N substitutions exhibited higher polymerase activity than PB2 derived from an avian type virus at both 33 and 37 °C in human embryonic kidney 293T (HEK293T) cells (Fig. 2a)^33^,^34^. Polymerase activity of the PB2 genotype with 526R was 10-fold higher than that of the avian-PB2 genotype, while two genotypes of PB2 carrying either 526R/627K or 526R/701N showed moderately increased and also consistently higher (two- to fivefold greater) polymerase activity than that associated with the single PB2 substitutions 627K or 701N. Notably, the enhanced effect of 526R in combination with 627K is consistently more apparent at 33 °C (Fig. 2a); this temperature is believed to favour the replication of influenza virus in the upper respiratory tract^8^. Because the minigenome system reporter assay mimics virus RNP in cells, any effect may be reflected in levels of viral messenger RNA (mRNA), viral RNA (vRNA) or protein. To understand whether the change in polymerase activity is related to differences in the levels of PB2 protein, we analysed viral proteins and found PB2 protein levels to be lower in the avian-PB2 group, but that there was no apparent difference between PB2-526R, PB2-701N, 526R/701N, PB2-627K and PB2-526R/627K in the minigenome assay (Fig. 2a). However, expression of FLAG-tagged versions of PB2 in the pCMV (cytomegalovirus) vector, which is not affected by PB2 polymerase properties, showed that avian PB2 has similar levels of PB2 protein. Hence, lower levels of PB2 protein in RNP containing avian PB2 are due to the low transcription and replication efficiency associated with avian PB2 and RNP polymerase complexes, as shown in subsequent experiments (Fig. 3a,b). In contrast to the result in HEK293T cells, the polymerase activity associated with avian type PB2 was not inhibited when avian cells (DF-1) were used in the RNP polymerase assay (Fig. 2e).

The seasonal H3N2 virus has been circulating continuously in humans since 1968, and is believed to have gained most of its adaptations through interaction with its human host. Since the 526R genotype was found among H3N2 viruses from around 1970 onwards and has become prevalent since then, we tested whether gaining 526R enhanced virus replication. Introduction of 526R into the PB2 of an H3N2 strain pre-dating this change (A/Hong Kong/1/68) significantly increased polymerase activity, which is consistent with the observations for H7N9 PB2 (Fig. 2b). Consistent with the effect of introduction of 526R into the 1968 H3N2 PB2 gene, reverse mutation of 526R to 526K in the PB2 of a recent H3N2 isolate (A/Guangdong/ST798/2008) led to decreased polymerase activity (Fig. 2c). We postulated that K526R may have provided additional optimization for the replication of the H3N2 virus following the incorporation of an avian PB1 into H2N2 in 1968 (ref. 35). To test this idea, we analysed the polymerase activity when 526R PB2 was combined with PB1 from the SG57 (A/Singapore/1/57, H2N2; original PB1) and Aichi68 (A/Aichi/2/68, H3N2; new avian-source PB1) viruses using a minigenome backbone derived from the 1957 virus. We found that replacement of PB1 in the SG57 RNP complex with A/Puerto Rico/8/1934 (PR8) PB1 resulted in decreased polymerase activity in repeated assays, suggesting introduction of PB1 requires compatibility with other subunits in the H2N2 RNP. However, incorporation of PB1 from Aichi68-H3N2 into the SG57 backbone, which mimics the formation of the H3N2 RNP as it occurred in 1968, does not compromise RNP polymerase activity. Introduction of 526R into the SG57-PB2 (627K) backbone enhanced polymerase activity. Furthermore, the RNP containing Aichi68-PB1 and 526R/627K PB2 consistently exerts higher polymerase activity than either whole SG57 or SG57 with Aichi68-PB1 in repeated experiments (Supplementary Fig. 2). Further studies are needed to unveil the role of PB2-526R in compatibility with PB1. Since 526R is predominantly present among H5N1 human isolates from Indonesia, we examined the effect of PB2-526R on polymerase activity in this subclade of the H5N1 subtype. It is notable that altering 526R to 526K in the PB2 completely abolishes RNP polymerase activity in the minigenome assay for RNP derived from A/Indonesia/5/2005 (Fig. 2d). These results clearly show that 526R, alone or coupled with 627K, provide an adaptive advantage to the polymerase complexes of avian H7N9 and H5N1 influenza A viruses during mammalian cell infection and optimizes RNP fitness during H3N2 virus replication.

### PB2-526R augments viral genome transcription and replication

To further understand whether the altered RNP polymerase activity and viral growth associated with 526R versions of PB2 is due to differences in the efficiency of virus genome transcription and replication, we examined levels of viral mRNA and vRNA in A549 cells infected with viruses containing different versions of PB2 at 6 h post infection. Quantitative reverse transcription-PCR (RT-PCR) analysis with mRNA- or vRNA-specific primers showed that A549 cells infected with viruses carrying either 627K, 701N or 526R PB2 have significantly higher levels of viral matrix protein (M1) and nucleoprotein (NP) mRNA and vRNA, as compared with cells infected with a virus containing avian PB2 (Fig. 3), suggesting these adaptation markers are critical for viral RNP transcription and replication in mammalian cells during the early phase of infection. Furthermore, addition of 526R to 627K or 701N provides an extra advantage in viral RNP transcription and replication efficiency. While the additional enhancement effect of 526R on 627K or 701N is not as strong as that seen when 627K is introduced into the avian PB2 genotype, the experiments were repeated four times, and consistently demonstrated that 526R exerts a positive effect on virus replication and transcription, either alone or when combined with 627K or 701N. It appears that the effect of 526R on viral genome transcription and replication is more apparent in the early hours of infection (Supplementary Fig. 3). These results suggest that 526R enhances the effects of 627K and 701N adaptations, resulting in optimal transcription and replication of influenza virus RNP in mammalian cells.

## 526R synergizes with other PB2 markers in H7N9 replication.

**Figure 4:**
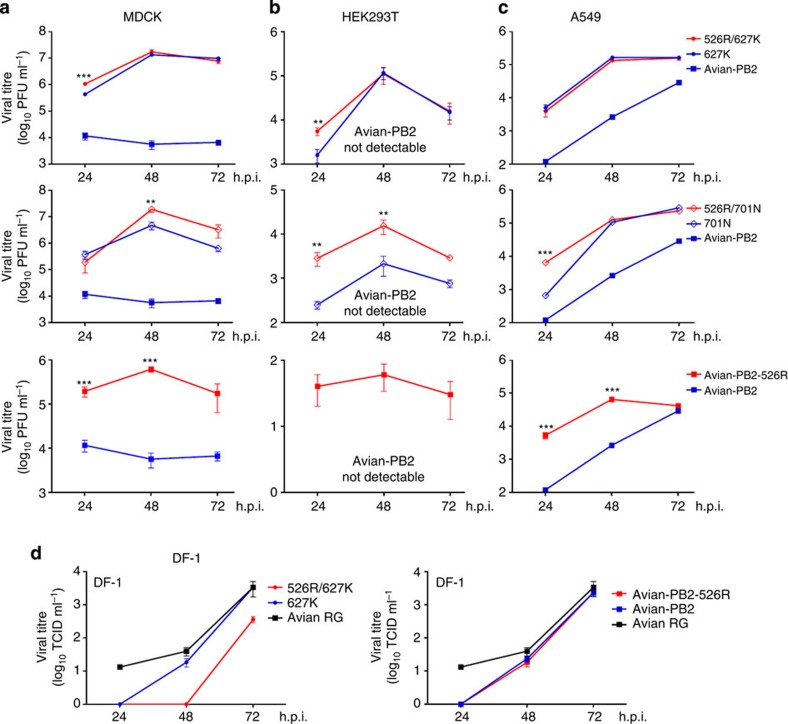
Growth kinetics of H7N9 viruses carrying different PB2 variants in mammalian and avian cells. Reverse genetic versions of H7N9 viruses containing avian-PB2, avian-PB2-526R, PB2-526R/627K, PB2-627K, PB2-526R/701N or PB2-701N, along with the remaining seven gene segments of A/Zhejiang/DTID-ZJU01/2013, were used to infect (**a**) MDCK cells at an MOI of 0.001 and (**b**) HEK293T cells and (**c**) A549 cells at an MOI of 0.01. The cells were cultured at 37 °C and supernatants collected at 24, 48 and 72 h post infection and subjected to plaque assays in MDCK cells to determine virus titres. (**d**) A subset of reverse genetic versions of H7N9 viruses containing avian-PB2, avian-PB2-526R, PB2-526R/627K or PB2-627K or rescued reverse genetic A/Chicken/Zhejiang/DTID-ZJU01/2013 (Avian RG), were used to infect DF-1 cells at an MOI of 0.01 and cells were then cultured at 39 °C. Supernatants were collected at 24, 48 and 72 h post infection and subjected to plaque assays in MDCK cells to determine virus titres. The values displayed are the log_10_ means±s.d. from three separate experiments. Statistical significance was analysed by one-way analysis of variance, corrected by the Bonferroni post-test: ***P<0.001 and **P<0.01. PFU, plaque-forming units; h.p.i., hours post infection.

To further characterize the phenotype of viruses carrying 526R PB2, reverse genetic (RG) versions of viruses from human isolates carrying the 627K, 701N, 526R/627K, 526R/701N and solely 526R PB2 genotypes were constructed in the A/Zhejiang/DTID-ZJU01/2013 backbone. Comparison of the growth kinetics of these viruses with a similarly constructed RG version of an avian H7N9 strain (A/Chicken/Zhejiang/DTID-ZJU01/2013) revealed that viruses carrying any of the adaptation markers, including the novel 526R, replicated to a 1–2 log higher titre than the avian H7N9 virus in Madin-Darby canine kidney (MDCK) cells (Fig. 4a). Our results showed that 526R significantly enhances replication of 627K virus at 24 h post infection, but that the enhancement is less apparent at the 48- and 72-h time points. One explanation may be that 526R is more critical during the early phase of virus replication in mammalian cells. Addition of 526R also significantly enhanced the growth rate of 701N virus. To further confirm the role of 526R in virus replication in other mammalian cells, we tested the same set of RG versions of H7N9 viruses in HEK293T and A549 cells, which are also commonly used for studying influenza virus infection. While an avian version of H7N9 viruses, with avian PB2 in a human virus backbone (avian PB2), is unable to replicate in HEK293T cells, other H7N9 viruses that contain different PB2 adaptation markers were able to replicate to various levels in both HEK293T and A549 cells, with the 526R/627K version of the virus replicating to the highest levels (Fig. 4b,c). In contrast to the results obtained in MDCK, HEK293T and A549 cells, there is no replication advantage in avian cells (DF-1) for viruses containing either single or coupled 526R/627K PB2 mammalian adaptation markers, with the 526R/627K genotype being inhibited (Fig. 4d). To exclude the possibility of underestimation of the growth of avian PB2 virus in the MDCK cell-based plaque assay, we also tested the titres of avian PB2 and avian 526R PB2 viruses by assessing the 50% egg infectious dose, with the results indicating that the plaque assay reflects the trend of virus titres obtained in the growth kinetics experiments. These results further confirmed that the PB2-526R substitution may enhance replication of H7N9 viruses in mammalian cells, but not in avian cells.

### PB2-526R enhances H7N9 virus replication in mice

**Figure 5:**
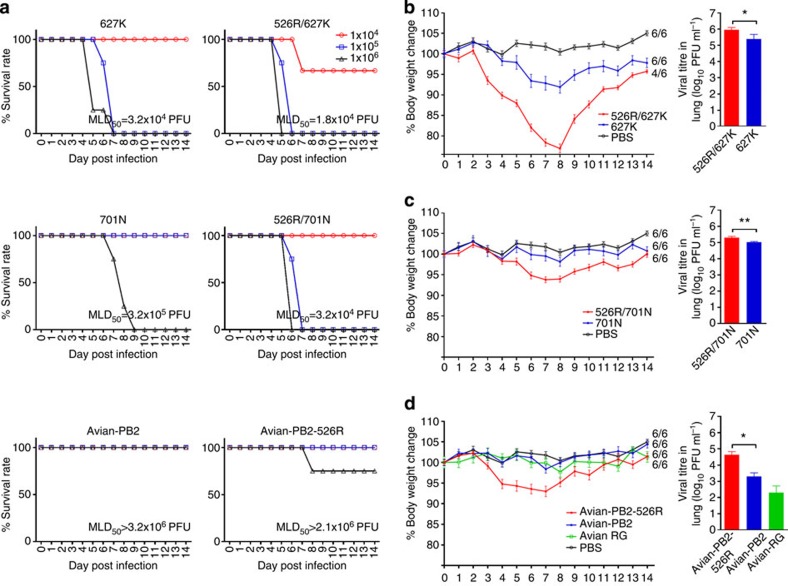
Infection and replication of H7N9 viruses with different PB2 genotypes in mice. (**a**) Groups of mice were infected with 25 μl inoculums containing 10^4^, 10^5^ or 10^6^ PFU of reverse genetic versions of viruses, as specified. Mice were observed daily for changes in body weight for 14 days (day 0 to day 14). Animals that lost >25% of their pre-infection weight were euthanized, in accordance with our institutional animal ethics guidelines. The MLD_50_ values were calculated by the method described by Reed and Muench^56^. To test virus replication in mice, groups of nine BALB/c mice were each inoculated intranasally with 2.25 × 10^4^ PFU (25 μl) of H7N9 recombinant virus containing PB2-526R/627K, PB2-627K, PB2-526R/701N, PB2-701N, avian-PB2, avian-PB2-526R or with a wholly reverse genetic version of avian H7N9 virus (avian RG). Results compare the mean percentage weight loss or gain for groups of mice infected with 627K or 526R/627K viruses or administered PBS (control) (**b**), 701N or 526R/701N viruses or PBS control (**c**) and avian RG, avian-PB2 or avian-PB2-526R viruses or PBS control (**d**); error bars represent s.d. The fractional number labels within the graphs indicate the number of surviving mice in each of the groups at the end of the experiment. Three mice from each of the groups infected with viruses carrying 627K or 526R/627K (**a**, right panel), 701N or 526R/701N (**b**, right panel) or with RG avian H7N9 virus, avian-PB2 or PB2-526R in the backbone of a human isolate (A/Zhejiang/DTID-ZJU01/2013) were killed at 72 h post infection. Lung tissues were removed and homogenated for estimation of virus replication by the plaque assay. The values displayed represent the log_10_ mean titres±s.d. Statistical significance was calculated by one-way analysis of variance, corrected by the Bonferroni post-test: **P<0.01 and *P<0.05. d.p.i., days post infection.

To further examine the effect of PB2-526R on virus replication, RG versions of H7N9 viruses representing the different PB2 genotypes identified in human isolates were tested in a mouse infection model. We first estimated the 50% mouse lethal dose (MLD_50_) of H7N9 viruses containing different genotypes of PB2. Our results showed that inclusion of K526R, alone or coupled with 627K or 701N, increased mortality in the infected mice (Fig. 5a). To confirm that the K526R substitution caused increased virus replication *in vivo*, mice were infected with a sub-lethal (2.25 × 10^4^ plaque-forming units (PFU), based on pre-experimental data obtained using either 627K or 701N PB2 genotype viruses) dose of one of the different versions of H7N9 virus and were observed, and changes in body weight recorded over the following 14 days. Viruses carrying 526R or 627K substitutions caused moderate body weight loss (Fig. 5b,d), while RG avian H7N9 viruses or viruses with 701N alone did not cause any apparent loss of body weight in infected mice, as compared with the phosphate-buffered saline (PBS) control or avian-type PB2 virus-infected groups (Fig. 5c,d). Consistent with the results of the RNP polymerase activity and growth kinetics assays, the 526R/627K PB2 genotype virus caused the greatest body weight loss in mice, which peaked at around 25% on day 8 post infection (Fig. 5b). To examine virus replication ability in mouse lung tissues, three mice from each of the PB2-526R, 526R/627K, 627K, 526R/701N, 701N, avian-PB2, avian-PB2-526R and pure avian H7N9 (avian RG) infection groups were euthanized on day 3 post infection and lung tissues collected for virus titration. Consistent with the body weight loss result, viruses carrying 526R/627K replicated to the highest titres in the lungs of mice, about 1.5 to 2 logs higher than that in the lungs of mice infected with H7N9 virus carrying avian type PB2 and significantly higher than virus with 627K alone (Fig. 5b,d). While 701N PB2 virus caused no apparent body weight loss in mice, it replicated to a reasonably high titre in the lungs of infected mice and its replication ability was enhanced by the addition of 526R (Fig. 5c). H7N9 viruses carrying 526R PB2 also exhibited enhanced replication (about 10-fold) in the lung tissues of infected mice (Fig. 5d). Comparison of virus titres from mice infected with 526R/627K, 526R/701N or 526R viruses mirrored the results of the RNP activity and growth kinetics analyses, confirming that 526R/627K provides the most optimal PB2 genotype for replication of H7N9 virus in mammalian cells. These results confirm that the PB2-526R adaptation optimizes the *in vivo* replication ability of H7N9 viruses in mammalian host species.

### PB2-526R confers a growth advantage in H3N2 virus

**Figure 6:**
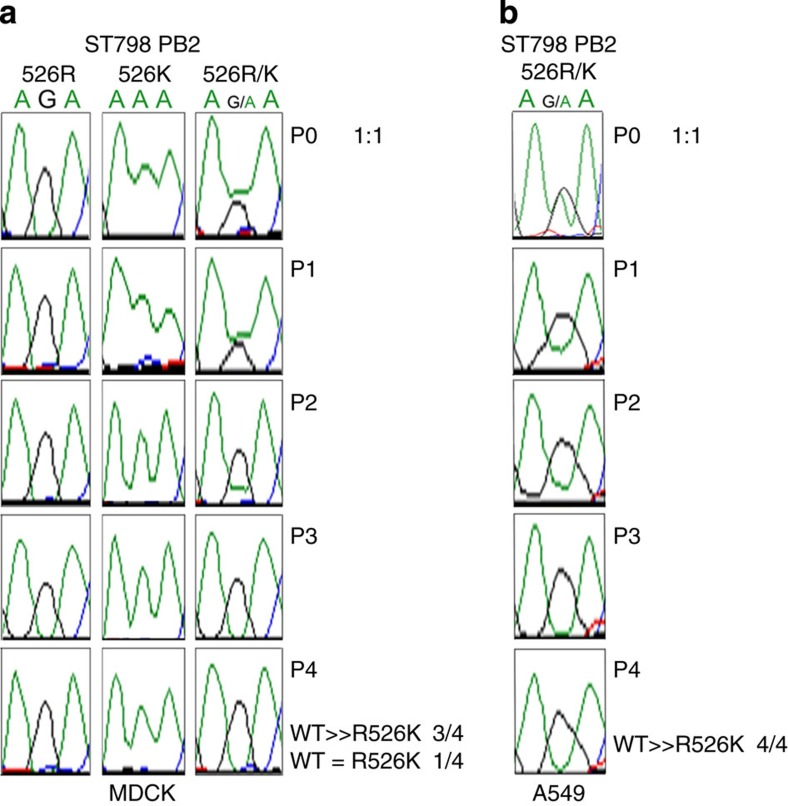
Comparison of the growth rate of H3N2 A/Guangdong/ST798/2008 strains carrying either 526R or 526K PB2 in mixed cultures. (**a**) MDCK cells or (**b**) A549 cells were infected at an MOI of 0.001 with a mixture (1:1) of wild-type (WT) (PB2-526R) and variant (PB2 R526K) A/Guangdong/ST798/2008 and sequentially passaged four times. At each passage viral RNA was isolated from cell culture supernatants at 48 h post infection, and the PB2 gene amplified by RT-PCR and sequenced. The genetic code for WT PB2-526R is AGA; variant PB2 526K is AAA. The DNA sequence chromatograms shown are from one of the three MDCK (**a**) and one of the four A549 (**b**) experiments in which 526R outgrew 526K. Experiments were repeated four times.

As described above, the H3N2 virus obtained K526R around 1970 and the PB2-526R genotype immediately became the dominant circulating H3N2 in humans, persisting to the current day (Fig. 1). We have shown that addition of 526R to the PB2 of A/Hong Kong/1/1968 H3N2 virus enhances RNP polymerase activity, while back-mutation of 526R to 526K in the PB2 of A/Guangdong/ST798/2008 decreased RNP polymerase activity (Fig. 2b,c). To test whether gaining 526R has provided an additional growth advantage to the H3N2 virus, we constructed RG versions of the H3N2 virus and a variant with a 526K back-mutation in the A/Guangdong/ST798/2008 backbone. We adopted an assay that was previously used for measuring a minor growth advantage of H3N2 virus, obtained through acquisition of seven C-terminal amino acids in influenza non-structural protein 1, around 1950 to measure the growth rate advantage associated with 526R PB2 (ref. 36). Evenly mixed 526R and 526K PB2 versions of H3N2 virus were used to infect MDCK and A549 cells and then passaged sequentially. Aliquots of virus were collected from each passage and subjected to sequence analysis of the PB2-526R/526K mixed populations. All four of the independent experiments performed showed that 526R PB2 virus had completely outgrown 526K at the 4th passage in A549 cells (4/4), while only one showed no change to the mixed populations in MDCK cells (3/4) (Fig. 6a,b). In contrast to the effect on H3N2 virus, introduction of K526R into PR8 (H1N1) virus compromised virus replication in a similar assay (Supplementary Fig. 4), suggesting that the dual 526R/627K adaptive strategy is RNP complex specific. This evidence supports our observations from H7N9 and H5N1-Indonesia viruses and indicates that the K526R substitution in the PB2 polymerase subunit provides a growth advantage to H3N2 influenza A virus, which may further enhance replication in humans.

### PB2-526R optimizes interaction between PB2 and NEP

**Figure 7:**
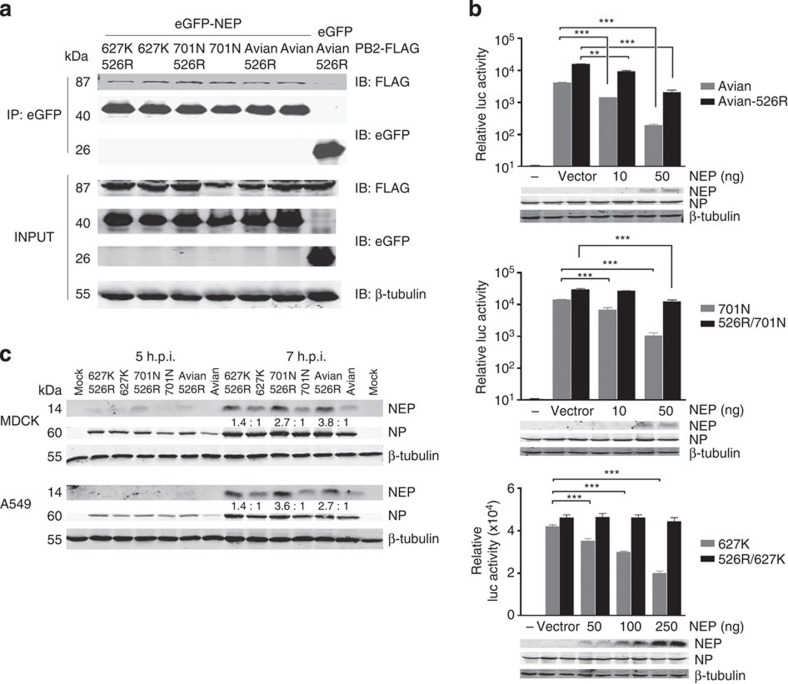
Effect of PB2-526R in interaction between PB2 and NEP. (**a**) Co-immunoprecipitation of NEP with different versions of PB2 polymerase derived from H7N9 virus. Enhanced GFP (eGFP)-NEP and FLAG-PB2 were co-expressed in HEK293T cells. Whole**-**cell extracts were used for immunoprecipitation with an anti-GFP antibody and analysed by western blot analysis, using specific antibodies against eGFP and FLAG. The experiment was repeated three times. (**b**) Effect of different levels of NEP on RNP polymerase activity. RNP complexes containing different versions of PB2 were co-transfected with increasing amounts of NEP (H7N9) expression vector or empty vector, together with the firefly luciferase reporter pYH-Luci and a *Renilla* luciferase reporter (internal control). Luciferase activity was measured at 24 h post transfection, following incubation at 37 °C, and the effect of different amounts of NEP on the polymerase activity of RNP with and without PB2-526R was compared. Data represent mean luciferase activity from three separate experiments, calculated after normalization with *Renilla* luciferase activity,±s.d. ‘—’ represents blank control; RNP without the PB2 gene. Statistical significance was analysed by the Student’s *t*-test: ***P<0.001, **P<0.01. (**c**) Western blot analysis of NEP expression in MDCK and A549 cells infected with H7N9 viruses carrying different versions of the PB2 segment. Confluent MDCK or A549 cells were infected with various reverse genetic H7N9 viruses, at an MOI of 1. Cells were collected at 5 and 7 h post infection and whole-cell lysates were analysed for NEP and NP expression by western blot using antibodies specific for NEP and NP, respectively. Blotting with antibody against β-tubulin, which served as a loading control, was also performed. Numbers indicate the ratio of NEP expression between viruses carrying 526R in the PB2 and those without 526R. The experiment was repeated twice. Full-size western blots are provided in Supplementary Fig. 6.

The molecular mechanism underlying host adaptation of influenza A virus through PB2 mutation remains largely undescribed. The viral NEP has been suggested to be involved in influenza virus host adaptation through its action as a regulator to switch on influenza virus genome transcription and replication^37^,^38^. Expression of NEP is regulated during virus replication, and increased expression of NEP brought about through disruption of splicing control is deleterious to virus replication^37^,^39^. We confirmed the interaction between PB2 and NEP in a co-immunoprecipitation assay using NEP and different versions of PB2 derived from H7N9 virus (Fig. 7a). To test the impact of co-expression of NEP on RNP polymerase activity, we co-expressed RNP containing various PB2 genotypes with increasing amounts of H7N9 virus NEP in HEK293T cells. Our result showed that increased levels of NEP exert an inhibitory effect and that this effect is much more profound for RNP with avian type PB2, but less so for RNP complexes containing PB2-526R, PB2-701N and PB2-627K. It is notable that when 526R is coupled with 627K in PB2, the resulting RNP complex is able to tolerate much higher levels of NEP (Fig. 7b). A similar inhibitory effect on RNP polymerase activity by avian type NEP has been observed for H5N1 viruses^37^. No adaptive substitutions have been observed in the NEP of H7N9 virus. Analysis of NEP expression in MDCK and A549 cells infected with H7N9 viruses carrying various PB2 markers confirmed that NEP is not expressed in the earliest hours of infection and that PB-526R-bearing viruses express higher levels of NEP than their counterparts that lack this substitution (Fig. 7c). Since PB2 interacts with both NP and PB1, we examined the PB2–NP interaction, and found that 526R enhances the interaction between PB2 and NP in the context of the RNP (Supplementary Fig. 5). While further studies are needed to reveal the molecular details of how PB2 and NEP interact with each other and host factors, it seems possible that the K526R-adaptive substitution enhances the interaction between PB2, the other RNP subunits and NEP during virus replication.

## Discussion

Historically, very few influenza viruses have become established in mammalian hosts, namely the H1N1, H2N2 and H3N2 subtypes. Host adaptation requires the virus to overcome restriction barriers and replicate efficiently in a new host. To overcome host barriers, viruses must gain specificity for binding to cell receptors, adapt to use host machinery for genome replication, transcription and protein synthesis in cells and evade restriction by elements of the host innate immune system during virus infection. The influenza virus replication complex is composed of three polymerase subunits (PB1, PB2 and PA) plus NP. Characterization of host adaptation markers among human isolates is important for recognizing potential for cross-species transmission in avian influenza A viruses. Adaptation of avian influenza A virus polymerases in mammals has been extensively studied and the most well-characterized adaptation markers are located in the PB2 polymerase subunit^40^. The PB2 E627K substitution is recognized as a dominant adaptation marker in the majority of human-adapted influenza A viruses, facilitating replication in mammalian cells, however, the 590S/591R motif was found to complement the function of 627K in the PB2 of 2009 H1N1 virus to allow replication in humans^41^. While the 627K marker and, to a lesser extent, the 701N substitution have been described as being critical for replication of avian H5N1 and H7N7 viruses in mammalian hosts^3^,^9^, no known PB2 adaptation marker has previously been characterized in the majority (~80%) of H5N1 human isolates from Indonesia^17^,^42^.

Human infections with H7N9 virus emerged in China in March 2013 (refs 19, 20, 31). While efficient human to human transmission was not observed and a serological study found no evidence of circulation of H7N9 virus in the general population^43^, sporadic inter-human transmissions were observed and laboratory transmission studies indicate that, unlike the H5N1 virus, this virus may be able to be transmitted between ferrets^44^,^45^,^46^,^47^,^48^. Genetic analysis showed that the H7N9 virus is currently still of the avian genotype^49^. In addition to the Q226L substitution in the HA, which may provide the virus with some ability to bind to human type receptors^26^,^27^, what other distinct properties has this virus gained to make it different from other avian influenza viruses? This study found that isolates from human H7N9 cases carried one or more adaptation markers in the PB2 polymerase gene (Table 1). No similar association was found among avian or environmental H7N9 isolates, suggesting these adaptive changes occurred after virus replication in human cells. A distinct substitution, K526R, was found among some of the human isolates, in conjunction with 627K or 701N markers. The K526R substitution has not been recognized as a mammalian adaptation marker previously. However, sequence surveillance found that while 526R is not commonly present in human H5N1 isolates from most countries, nearly 80% of isolates from H5N1 human cases identified in Indonesia contain this marker (Table 1). It seems likely that different genetic lineages of avian influenza viruses evolve by using different adaptive pathways to enable their replication in humans. For example, the 2009 H1N1 virus adapted by using a PB2 590S/591R motif that synergized with the function of 627K to gain the ability to replicate in humans. A similar adaptive strategy was found among H9N2 human isolates, with 7 out of 10 human isolates containing the 590S substitution (Table 1).

The 526R PB2 mutation is rarely found among avian isolates and it was logical to presume that it may be an uncharacterized marker for human adaptation. Importantly, we found that the H3N2 virus has also gained the PB2-K526R substitution, in addition to 627K; this occurred around 1970, based on available sequence data, and this genotype has become prevalent in humans since then (Table 1; Fig. 1). It may be speculated that the introduction of an avian PB1 into the H2N2 virus in the 1968 reassortment event might have necessitated a requirement for rebalancing of RNP fitness in the H3N2 virus, with the combination of 526R/627K providing optimal efficiency for RNP polymerases during H3N2 virus infections in humans. Indeed, our results showed that PB2-526R/627K elicits higher RNP polymerase activity when combined with the PB1 from 1968 reassortant H3N2 virus than does PB2-627K (Supplementary Fig. 2). Introduction of 526R or 526K substitutions into A/Hong Kong/1/1968 (526K PB2) and A/Guangdong/ST798/2008 (526R PB2) PB2 proteins led to enhanced and reduced RNP polymerase activity, respectively, in minigenome assays (Fig. 2). Furthermore, the A/Guangdong/ST798/2008 version of H3N2 virus carrying 526R PB2 completely outgrows the 526K variant after only four passages in infected MDCK and A549 cells (Fig. 6). It remains to be tested whether the frequently higher seasonal activity of H3N2 virus in humans, compared with H1N1, may be due to its more optimal replication properties and if gaining K526R has contributed to the fitness of H3N2 virus. Avian H5N1 virus has caused >600 human infections in over a dozen countries since 1997, and various adaptation markers in PB2 were observed among human isolates (Table 1). It was suggested that the effect of 627K on H5N1 virus may be dependent on the virus lineage^18^. Interestingly, PB2 carrying 526R, but not 627K, is commonly found among H5N1 human isolates from Indonesia, which has the highest number of human H5N1 cases and the highest mortality rate to date. We showed that mutation of PB2-526R to 526K completely abolished polymerase activity for RNP complexes derived from one of the human isolates, A/Indonesia/5/2005 (Fig. 2d). Taken together, these results support the notion that K526R is a new adaptation marker for H7N9 and H5N1-(Indonesia) avian influenza A virus in humans and also provides an extra advantage for H3N2 virus replication.

Protein structure data for the C-terminal domain of PB2, which contains the 627 and 701 residues, suggests that either 627K or 701N may enhance PB2 exposure to allow better interaction with elements of the host machinery such as importins, which facilitate nuclear import during virus infection^50^,^51^. There is no structural data available for analysing the domain that covers the region containing residue 526. How this region may enhance the fitness of PB2, or whether it interacts with residues 627 and 701 or other polymerase subunits, remains to be determined. Recent studies suggest that NEP has a role in regulating influenza A virus genome transcription and replication, independent to its nuclear export function^37^. This study provides preliminary evidence that gaining the 526R substitution in the PB2, in addition to other adaptation markers, could enhance RNP polymerase activity through coordination with NEP expression for more efficient viral transcription and genome replication during influenza A virus infection.

Further studies are necessary to determine whether viruses that have gained dual 526R/627K or 526R/701N substitutions may transmit more efficiently in humans, or in animal models. Full evaluation of the transmission and replication abilities of H7N9 and other avian influenza A viruses, such as H5N1-(Indonesia), bearing K526R PB2 will provide valuable information for understanding the adaptation process and establish the importance of this new marker in surveillance of zoonotic influenza A viruses.

## Methods

### Viruses

All influenza viruses used in this study were rescued using the RGs technique to ensure the absence of contamination from other microbes. Viruses were propagated in embryonated chicken eggs (9–11 days old) for 3 days (P1), using the standard protocol approved by the Committee on the Use of Live Animals in Teaching & Research (CULATR) of the University of Hong Kong.

### Viral genome characterization and RGs

The H7N9 viruses from laboratory-confirmed human cases have been characterized in our previous studies^20^,^52^. The sequences of strains of H7N9 and H3N2 viruses used in this study have been deposited in Genbank (Accession codes AGU02226 to AGU02234, KJ633805, and KJ473719 to KJ473726). Eight segments from the A/Zhejiang/DTID-ZJU01/2013 and A/Chicken/Zhejiang/DTID-ZJU01/2013 strains were cloned into a pHW2000 plasmid system^33^,^53^,^54^, to be used as backbones. RG versions of both A/Zhejiang/DTID-ZJU01/2013 (human, ZJ/1, PB2-701N) and A/Chicken/Zhejiang/DTID-ZJU01/2013 (avian RG) were constructed. The ZJ/1 backbone was used to rescue recombinant viruses with various PB2 genes containing different adaptation markers. Minigenomes from the H3N2 strains A/Hong Kong/1/68 and A/Guangdong/ST798/2008, the H5N1 strain A/Indonesia/5/2005 and RG versions of A/Guangdong/ST798/2008 were cloned similarly and used to rescue virus together with 526K or 526R PB2 genes. Additional PB2 variants were generated using the QuikChange mutagenesis kit (Agilent) and also rescued using the ZJ/1 backbone. Full-length PB2 sequences of human and avian H5N1, H9N2, H7N7 and H7N9 viruses from the National Center for Biotechnology Information (NCBI) Influenza Virus Resource Database, together with all influenza A H7N9 human isolates in the Global Initiative on Sharing All Influenza Data (GISAID) database (http://gisaid.org) were analysed for prevalence of the K526R substitution.

### Minigenome reporter assays

Luciferase activity-based minigenome reporter assays were performed as described previously^53^. RNP complexes composed of PA, PB1, PB2 and NP derived from A/Zhejiang/DTID-ZJU01/2013 and cloned into the pHW2000 vector (50 ng each) were mixed with a luciferase reporter plasmid (50 ng) and a thymidine kinase promoter-*Renilla* luciferase reporter plasmid (pRL_TK) construct (10 ng), then co-transfected into HEK293T cells and incubated at 37 °C or 33 °C, supplied with CO_2_. For luciferase reporter assays in DF-1 cells, a luciferase reporter containing a chicken RNA polymerase I (Pol I)-driven promoter was constructed based on the sequence previously described^55^, and the transfected DF-1 cells were incubated at 39 °C. The luciferase activity was measured using a Dual-Luciferase Reporter Assay System (Promega) at 24 h post transfection. RNP polymerase activity was normalized against pRL_TK activity. To test the effect of NEP on RNP activity, experiments were performed as previously described^37^. The coding region of the RNP complex (PB1, PB2, PA and NP) and NEP were cloned separately into the pCX plasmid. HEK293T cells were transfected with the pCX-based RNP expression plasmids, the NEP expression plasmid and the two luciferase reporter plasmids, and RNP activity measured at 24 h post transfection.

### Growth kinetics of virus in cells

Confluent MDCK cells were infected with RG viruses at a multiplicity of infection (MOI) of 0.001. The viral inoculums were removed after 1 h of adsorption at 37 °C and replaced with minimal essential media (MEM) containing 1 μg ml^−1^ of L-(tosylamido-2-phenyl) ethyl chloromethyl ketone (TPCK)-treated trypsin. Infected cells were further incubated at 37 °C, supplied with 5% CO_2_. Culture supernatants were collected at different time points post infection and viral titres determined by the plaque assay in MDCK cells. Confluent DF-1 cells were infected with RG viruses at an MOI of 0.01. The viral inoculums were removed after 1 hours’ adsorption at 39 °C, then cells were washed with MEM and overlaid with MEM containing 0.5 μg ml^−1^ of TPCK–trypsin. Infected cells were further incubated at 39 °C, supplied with 5% CO_2_. Culture supernatants were collected at different time points post infection and viral titres determined by the standard 50% tissue culture infective dose assay in MDCK cells.

### Replication in mice and lethal dose determination

Female BALB/c mice, aged 4–6 weeks, were obtained from the Laboratory Animal Unit, the University of Hong Kong. For MLD_50_ determination, groups of four or six mice were anesthetized with isoflurane (Halocarbon Laboratory), and intranasally inoculated with 25 μl of 10-fold serial dilutions of RG virus in PBS, the dose range being 10^4^–10^6^ PFU. Body weight and survival were monitored daily for 14 days after infection. The MLD_50_ of RG viruses were calculated by the method of Reed and Muench^56^. To test virus replication in mice, groups of six or nine mice were anesthetized with isoflurane, and inoculated intranasally with 2.25 × 10^4^ PFU (25 μl) of H7N9 RG viruses. Animals were observed daily for mortality and body weight was measured for up to 14 days after infection. At 72 h post infection, three mice from each of the groups infected with virus containing 526R/701N, 701N, 526R/627K or 627K PB2, avian-PB2, avian-PB2-526R or with RG avian H7N9 virus, or mock-infected with PBS were euthanized and lung tissues were collected from each mouse for virus titration and histochemical staining. The protocols for animal experiments and the use of embryonated chicken eggs (9–11 days old) were approved by the CULATR, Li Ka Shing Faculty of Medicine, the University of Hong Kong (CULATR 3064-13). The CULATR follows the Hong Kong legislation and standards/guidelines recommended by the Association for Assessment and Accreditation of Laboratory Animal Care International (http://www.aaalac.org/about/guidelines.cfm).

### Quantitative RT-PCR assays for vRNA and mRNA

To test the specificity of the qPCR assay for viral mRNA, an NP-expressing plasmid was transfected into HEK293T cells for 48 h. Total RNA was extracted using the RNAiso reagent (Takara). Plasmid DNA present in the total RNA was eliminated by deoxyribonuclease (DNase) treatment (Ambion), in accordance with the user manual. Purified viral particles were used to test the specificity of the qPCR assay for vRNA. Total vRNA was again extracted using RNAiso. Complementary DNA was synthesized using oligo dT, uni-12 or uni-13 primers, and the copy number of NP was then quantified by qPCR using NP-specific primers.

To evaluate the levels of vRNA and mRNA in virus-infected cells, A549 cells were infected with different H7N9 viruses at an MOI of 1. Total RNA was extracted at selected time points. One microgram of total RNA was reverse transcribed using a high-capacity cDNA synthesis kit (Invitrogen). For detection of vRNA and mRNA, uni-12 primer and oligo dT primer, respectively, were used in the RT reaction. Expression of these two RNA species of the NP and M1 genes was quantified using SYBR Premix Ex Taq reagent (Takara) in an ABI 7500 real-time PCR machine (Applied Biosystems Inc.). For the detection of the M1 gene, M1-72s (5′-cgcacagagacttgaggatg-3′) and M1-270as (5′-tgggtctccattcccattta-3′) primers were used. For the detection of NP, NP-545s (5′-cagtgaaggggatagggaca-3′) and NP-746as (5′-ccaggatttctgctctctcg-3′) primers were used. β-actin expression was detected for each sample and used for normalization of gene expression between different samples. The amplification programme was as follows: 95 °C for 30 s, followed by 40 cycles of 95 °C for 5 s, 60 °C for 30 s. The specificity of the assay was confirmed by melting-curve analysis at the end of the amplification programme (65 to 95 °C, 0.1 °C s^−1^) (ref. 57).

### Sequential passage of mixtures of H3N2 PB2-526R and 526K

Mutation at the PB2 526 position was performed using the QuikChange site-directed mutagenesis kit (Agilent) and RG H3N2 wild-type and PB2 mutant viruses were rescued, as previously described^54^. Passage of H3N2 A/Guangdong/ST798/2008 wild-type virus (PB2-526R) and its mutant (PB2-526K) was carried out according to established procedures^36^. MDCK and A549 cells were infected at an MOI of 0.001 with wild-type ST978 virus, the mutant virus or a 1:1 mixture of the two viruses. Four serial passages were performed, each with an MOI of 0.001. vRNA was isolated from cell culture supernatants at 48 h post infection, and the PB2 gene amplified by RT-PCR and sequenced.

### Immunoprecipitation assay

To study the interaction between NEP and PB2, HEK293T cells were transfected with green fluorescent protein (GFP)-tagged NEP expression plasmid and FLAG-tagged PB2 expression plasmid. After 48 h, cells were lysed in cell lysis buffer (Tris-HCl 50 mM, 150 mM NaCl, 1% TritonX, pH 7.4). Cell debris was cleared by centrifugation, and 1 μl of GFP antibody (Invitrogen) was added to the cell lysate, which was then incubated for 3 h at 4 °C. Thirty microliters of protein A sepharose beads (GE Health) were then added to the lysate, followed by incubation for 1 h at 4 °C. The beads were washed with cell lysis buffer and finally boiled in SDS buffer at 95 °C. Proteins were resolved with SDS–polyacrylamide gel electrophoresis and detected by western blot using anti-GFP (dilution 1:1,000) (Invitrogen), anti-FLAG (dilution 1:3,000) and anti-β-actin (dilution 1:5,000) (Sigma) antibodies. To study the interaction between NP and PB2 in RNP complexes, pCX plasmids expressing PB1, PA, NP and FLAG-tagged PB2 were co-transfected into HEK293T cells. The immunoprecipitation steps were performed as described above, except that a laboratory-made polyclonal NP antibody (dilution 1:3,000) was used.

### Statistical analysis

Statistical analysis was carried out using GraphPad Prism V5 software (GraphPad Software Inc.).

## Author contributions

W.S. and P.W. performed the research, analysed and interpreted the results and wrote the manuscript. B.W.-Y.M., S.-Y.L., X.H., W.-L.W., M.Z., X.W., S.Y. and Y.C. performed animal experiments, virus isolation and analysed data. L.L. and K.-Y.Y. co-directed the study. H.C. directed the study, analysed and interpreted results and wrote the manuscript.

## Additional information

**How to cite this article:** Song, W. *et al.* The K526R substitution in viral protein PB2 enhances the effects of E627K on influenza virus replication. *Nat. Commun.* 5:5509 doi: 10.1038/ncomms6509 (2014).

**Accession codes:** Nucleotide sequences for the H7N9 and H3N2 viruses used in this study have been deposited in the NCBI GenBank database with accession codes AGU02226 to AGU02234, KJ633805, and KJ473719 to KJ473726.

## Supplementary Material

Supplementary Information Supplementary Figures 1-6

## Figures and Tables

**Table 1:** PB2 proposed mammalian adaptation markers among human and avian H5N1, H9N2, H7N9 and seasonal H3N2 isolates*.

**PB2 genotype**		**All H5N1**				**Indonesia H5N1**				**H9N2**				**H7N9**				**H3N2** †			
		**Human** ‡ **(** ***n*** **=261)**		**Avian** § **(** ***n*** **=1,290)**		**Human (** ***n*** **=70)**		**Avian (** ***n*** **=48)**		**Human** || **(** ***n*** **=8)**		**Avian** ¶ **(** ***n*** **=620)**		**Human** # **(** ***n*** **=96)**		**Avian (** ***n*** **=47)**		**1968–71 (** ***n*** **=90)**		**1972–2014 (** ***n*** **=4,872)**	
Proposed human adaptation marker	526R	47	194 (74%)	20	342 (27%)	47	56 (80%)	13	13 (27%)	1	7 (88%)	9	207 (33%)	4	81 (84%)	0	3 (6%)	0	90 (100%)	8	4,858 (99.7%)
	590S	8		38		0		0		5		192		1		0		0		0	
	591K	3		0		2		0		0		2		5		0		0		0	
	627K	114		279		1		0		0		4		61		3		66		17	
	701N	14		2		0		0		1		0		8		0		0		2	
	526R/627K	8		3		6		0		0		0		2		0		24		4,831	
Avian genotype**		67 (26%)		948 (73%)		14 (20%)		35 (73%)		1 (12%)		413 (67%)		15 (16%)		44 (94%)		0		14 (0.3%)	

^*^Full-length PB2 sequences of seasonal H3N2, human and avian H5N1, H9N2 and H7N9 influenza A viruses from NCBI Influenza Virus Resource Database, and human HPAI H5N1 from Influenza Sequence Database were analysed. Right column under each Human/Avian represents total number and percentage of isolates containing adaptation marker.

^†^Five human H3N2 isolates contains 526R/590S and one has 591K/701N.

^‡^Two human H5N1 isolates from Indonesia contain 526R/591K; two contain 590S/627K; two are 590S/701N and one contains both 627K and 701N markers.

^§^Two avian H5N1 isolates have 627K/701N and five contain 590S/627K.

^||^One human H9N2 isolate has triple adaptation markers, 526R/590S/701N.

^¶^Three avian H9N2 isolates contain dual 590S/591K signatures and one has 590S/627K.

^#^Three human H7N9 isolates contain 526R/701N genotypes.

^**^Avian genotype indicates isolates that lack any proposed human adaptation markers in the PB2.
