# Supplementary material for: The K526R substitution in viral protein PB2 enhances the effects of E627K on influenza virus replication
Source: Nat Commun. 2014 Nov 20;5:5509. doi: 10.1038/ncomms6509 (PMC4263149; doi:10.1038/ncomms6509)
Supplement: Supplementary Information — Supplementary Figures 1-6 [file ncomms6509-s1.pdf]

Supplementary materials

Supplementary Fig. 1

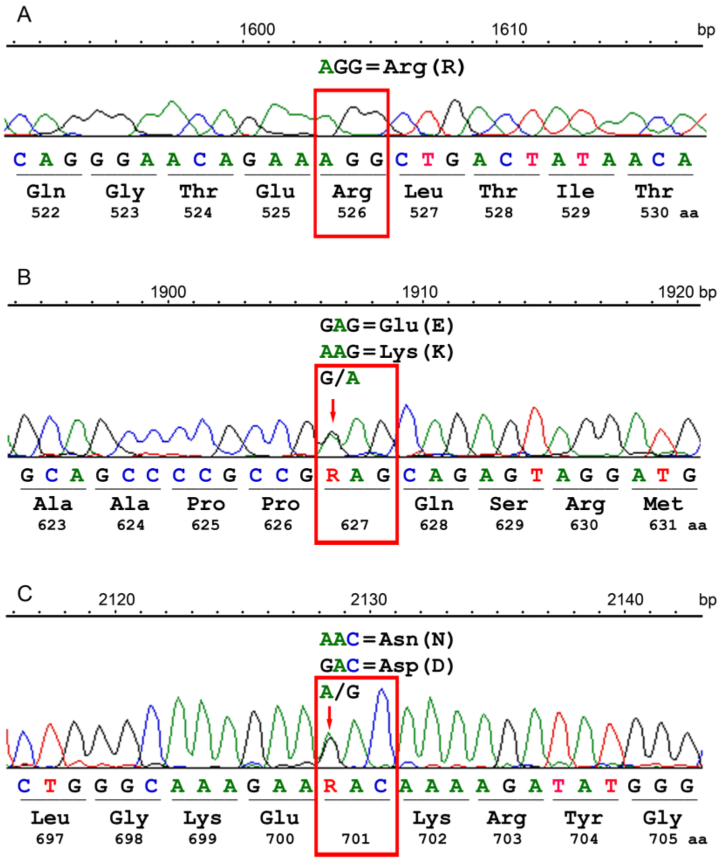

Supplementary Figure 1

Sequence chromatograms for the PB2 segment from A/Zhejiang/DTID-ZJU02/2013.

(A) The PB2 segment of the A/Zhejiang/DTID-ZJU02/2013 (ZJ/2) isolate contained an AAG to AGG substitution at nucleotide position 1603-1605, which encoded a change from a Lysine (K) to an Arginine (R) residue at amino acid position 526. (B) The sequence chromatogram shows the presence of a mixed population of G and A at nucleotide position 1906, leading to quasi species of PB2 627E and 627K, respectively. (C) Sequence chromatogram showing a mixed population of A and G at nucleotide position 2128, leading to the presence of quasi species of PB2 701N and 701D, respectively. Molecular cloning analysis showed that all of the PB2 genes were either 627E/701N or 627K/701D. The ratio of 627E/701N to 627K/701D is approximately 1:1.

Supplementary Fig. 2

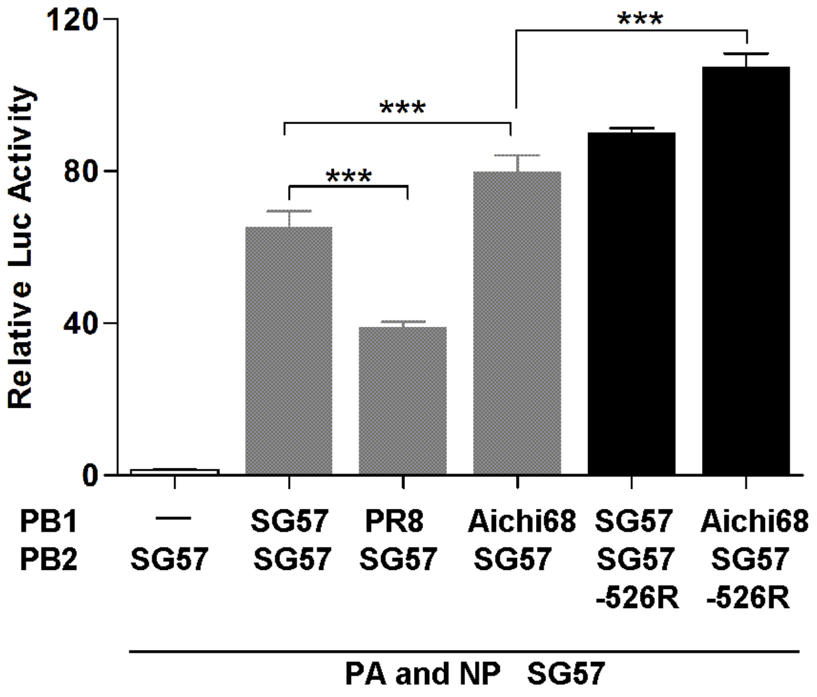

Supplementary Figure 2

Effect of PB2-526R on polymerase activity of PB1 from H2N2 and H3N2 viruses *in vitro*.

HEK293T cells were transfected with expression vectors containing PA and NP derived from A/Singapore/1/1957 (H2N2), and different versions of PB1 and PB2, as indicated, together with the firefly luciferase reporter pYH-luci and a *Renilla* luciferase reporter as a normalization control. Luciferase activity was measured at 48 hours post-transfection, following incubation at 37°C. Data represent mean luciferase activity from three separate experiments, calculated after normalization with *Renilla* luciferase activity, ± s.d. "-" represents blank control; RNP without the PB2 gene. Statistical significance was analyzed by one-way ANOVA, corrected by Bonferroni posttest: *** *P<0.001*.

Supplementary Fig. 3

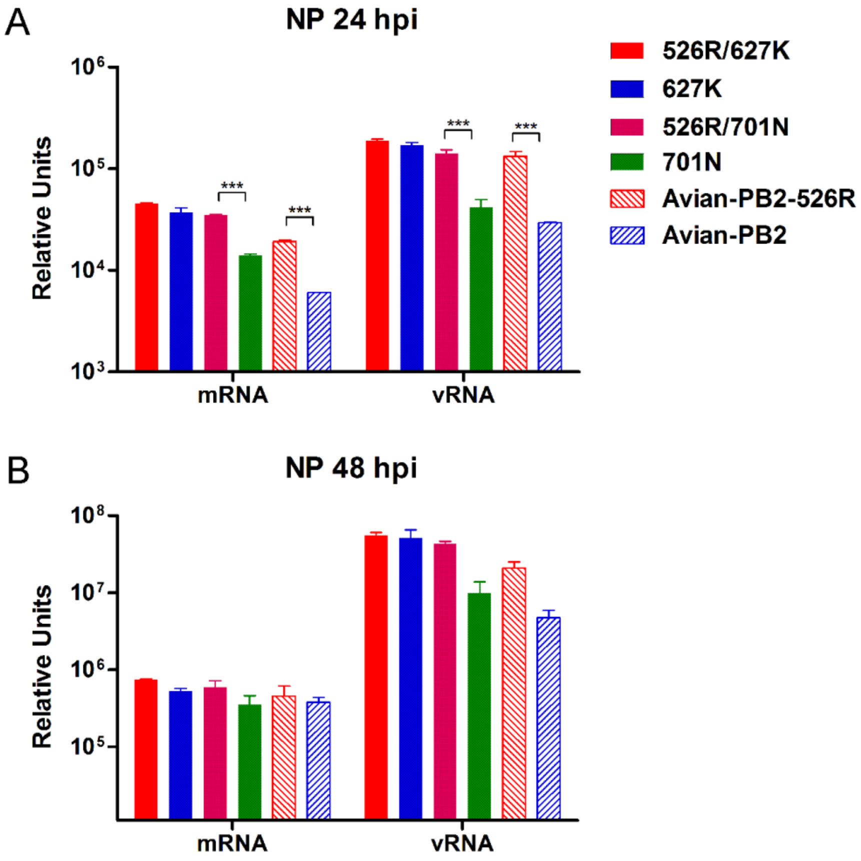

Supplementary Figure 3

Relative quantitative estimation of NP vRNA and mRNA levels in H7N9 virus infected A549 cells.

A549 cells were infected with H7N9 viruses carrying different PB2 adaptation markers, as indicated, at an MOI of 0.01. Uni-12 and oligo dT primers were used for reverse transcription of vRNA and mRNA, respectively. Levels of NP genes at 24 hpi (A) and 48 hpi (B) were estimated by relative quantitative RT-PCR and normalized with the *β-actin* gene, as previously described [57]. The values displayed represent the mean relative NP copy number ± s.d. from three separate experiments. Statistical significance was calculated by t-test: *** *P<0.001*, hpi: hours post infection.

Supplementary Fig. 4

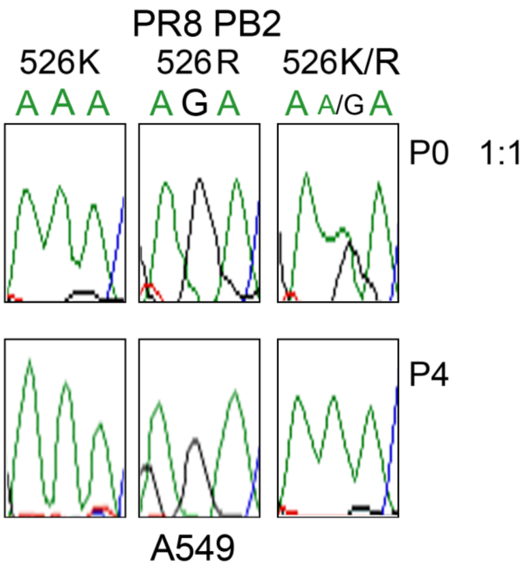

Supplementary Figure 4

Comparison of the growth rate of A/Puerto Rico/8/1934 (H1N1) strains carrying either PB2 526K or PR2 526R in mixed cultures.

A549 or MDCK cells were infected with WT A/Puerto Rico/8/1934 (H1N1) (PR8) (PB2-526K), mutant PR8 (PB2-526R), or a mixture of both strains (1:1) of at an MOI of 0.001. Cultures were passaged and viral RNA was isolated from cell culture supernatants after four passages, and the PB2 gene amplified by RT-PCR and sequenced. The genetic code for WT PB2 526K is AAA and variant PB2 526R is AGA. The sequence chromatograms are from one of the three sets of repeated experiments which showed similar outcomes. In the mixed culture, it appears that WT PR8-PB2-526K outgrew mutant PR8-PB2-526R.

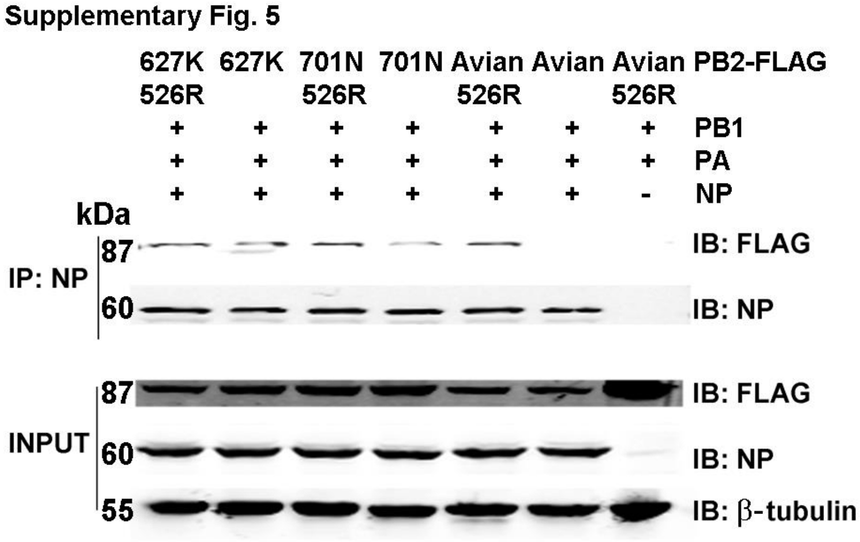

Supplementary Figure 5

Effect of PB2 adaptation markers on interaction with the NP protein.

Expression vectors containing PB1, PA, NP and different genotypes of C-terminal FLAG-tagged PB2 were transiently transfected into HEK293T cells for 24 hours. Whole cell extracts were prepared and used for immunoprecipitation with NP antibody, then proteins identified by western blot analysis, using specific antibodies against NP and the FLAG-tag. The experiment was repeated three times. Uncropped Western blots are shown in Supplementary Figure 6.

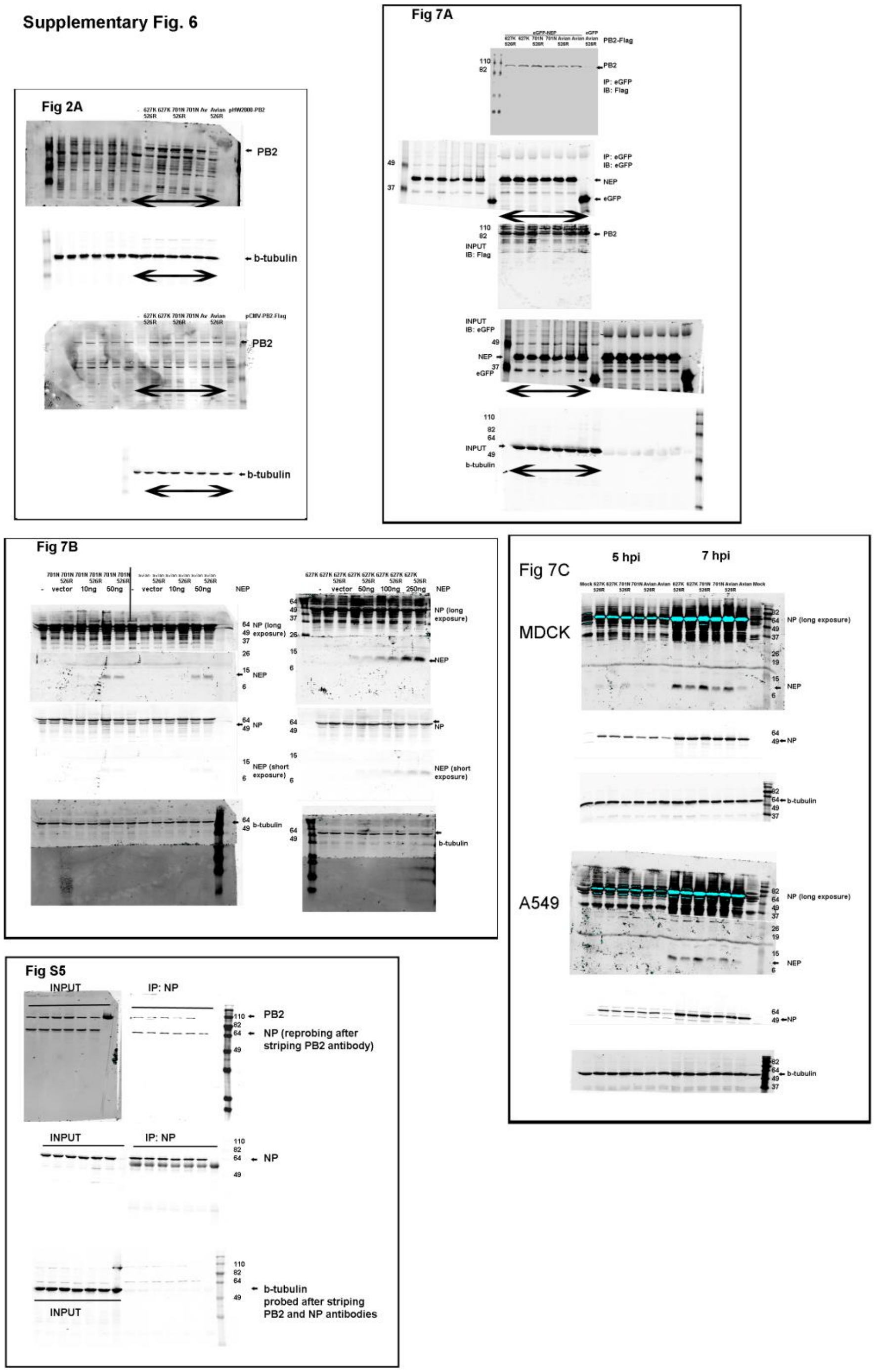

Supplementary Figure 6

Uncropped full size Western blot analyses.

Full sized Western blot analyses for results displayed in Figure 2, Figure 7 and Supplementary Figure 5.
